# Primary shoulder arthroplasty trends in Sweden: a 16-year observational study from 2008 to 2023

**DOI:** 10.1186/s12891-026-10201-8

**Published:** 2026-07-13

**Authors:** Ebrahim Oro, Viktor Mili-Schmidt, Michael Axenhus

**Affiliations:** 1 Department of Orthopaedic Surgery, Danderyd Hospital, Stockholm, Sweden; 2 Department of Clinical Sciences at Danderyd Hospital, Karolinska Institutet, Stockholm, Sweden; 3 Orthopaedic clinic, Danderyd University Hospital, Entrévägen 2 182 68, Danderyd, Sweden

**Keywords:** Incidence, Primary shoulder arthroplasty, Surgery, Sweden

## Abstract

**Background:**

Primaryshoulder arthroplasty is increasingly used to treat degenerative and traumatic shoulder conditions. This study aims to analyze incidence trends of primary shoulder arthroplasty in Sweden from 2008 to 2023 and to evaluate temporal changes in surgical and fixation methods, with projections for future incidence rates through 2025 and 2030.

**Methods:**

A retrospective, population-based study combining data from the Swedish National Patient Register (NPR) and the Swedish Shoulder Arthroplasty Register (SSAR). All patients aged ≥ 15 years undergoing primary shoulder arthroplasty between January 1, 2008 and December 31, 2023 were included. Incidence rates were calculated per 100,000 inhabitants, and regression modelling based on best fit were used to assess temporal trends and projections through 2030.

**Results:**

28,632 primary shoulder arthroplasties were performed (women: 18,569; men: 10,063). Overall incidence increased from 13.2 to 32.7 per 100,000. Women had higher increases in absolute incidence rates (17.4 to 41.2) while men showed a greater relative increase (8.8 to 24.2). HA declined steadily, becoming the least used method. Usage of RTSA increased, becoming the most common method in individuals aged ≥ 65 years. Cemented fixation declined across both age groups.

**Conclusion:**

The incidence of primary shoulder arthroplasty in Sweden more than doubled between 2008 and 2023, with a shift towards RTSA, away from HA and cemented fixation. Projections indicate continued growth through 2030.

## Background

Shoulder osteoarthritis is a painful condition that can cause loss of function and reduced quality of life [1, 2]. It is the most common indication for primary shoulder arthroplasty, followed by rotator cuff arthropathy and fractures [3]. The incidence of primary shoulder arthroplasty varies by sex and age. The highest rates are observed in older individuals and women undergo more procedures compared to men [4, 5].

Primary shoulder arthroplasty includes total shoulder arthroplasty (TSA), reverse shoulder arthroplasty (RTSA), and hemiarthroplasty (HA) [3]. Over the past decades, surgical practice has evolved. Usage of HA has steadily declined whilst RTSA and TSA are becoming increasingly more common [4, 6, 7]. Overall, incidence of primary shoulder arthroplasty is increasing world-wide [3, 7].

The Nordic countries have established national shoulder arthroplasty registries with prospective data collection, and several studies based on these registries have reported trends in utilization and outcomes. In Sweden, the NPR serves as the national administrative database with mandatory reporting from all healthcare providers, with complete nationwide coverage of all procedures [8–10].

The primary aim of this study was to describe the incidence of primary shoulder arthroplasty in Sweden from 2008 to 2023, stratified by age and sex. Secondary aims were to analyze temporal trends in surgical method and fixation technique, and to project future incidence rates through 2025 and 2030.

## Materials and methods

### Study design

This retrospective, observational population-based study is based on data from the Swedish National Patient Register (NPR) and the Swedish Shoulder Arthroplasty Register (SSAR).

### Setting

The Swedish National Health Service, administered by the Swedish National Board of Health and Welfare (SNBHW), provides universal healthcare to all citizens, encompassing emergency care, hospital treatments, and outpatient services. Every Swedish resident is assigned a unique personal identification number, which is utilized in all interactions with public or private healthcare and is recorded in all national healthcare registers.

### Data source

The NPR includes nationwide data on all specialized inpatient and outpatient care in Sweden. Reporting is mandatory for both public and private healthcare providers. The registry contains diagnosis codes based on ICD-10 and surgical procedure codes according to the Nordic Medico-Statistical Committee (NOMESCO) classification [11], as well as demographic information including age and sex. Complete nationwide coverage of surgical procedures from both inpatient and specialized outpatient care has been available since 2008; therefore, this year was chosen as the start of the study period. NOMESCO codes distinguishing RTSA from TSA were not implemented in the NPR until January 2018.

The SSAR is a national clinical quality register that prospectively collects data on shoulder arthroplasties from all hospitals performing these procedures in Sweden. The register records arthroplasty type, fixation, and indication per operation. Procedures are coded according to NOMESCO, with reverse total shoulder arthroplasty coded separately for the full study period 2008–2023.

### Patients

This study includes all patients ≥ 15 years at the time of surgery who have undergone shoulder arthroplasty between January 1st 2008 and December 31st 2023. The procedures included from the patient registry were coded according to NOMESCO [11]. All methods of shoulder arthroplasty were included; TSA (NBB29, NBB39, NBB49), RTSA (NBB59, NBB69), and HA (NBB09, NBB19). Implants used cemented (NBB19, NBB49), uncemented (NBB09, NBB29, NBB59) or hybrid (NBB39, NBB69) fixation. Revision arthroplasties were not included in this study.

### Variables

Variables included were year of surgery, age, sex, surgical method and fixation. Age was divided into two groups, individuals aged 15–64 and ≥ 65. The incidence is described as the number of procedures per 100,000 residents.

### Statistical analysis

Incidence rates were calculated per 100,000 inhabitants, with population estimates sourced from Statistics Sweden for each corresponding year. Exponential, linear, logarithmic, polynomial and Poisson regression models was used to assess temporal trends and predict future incidence rates. Predictive analyses were performed on the best fitting model for each incidence trend. Incidence rates were stratified by sex and age. Descriptive statistics were used to summarize patient demographics and surgical details. Confidence intervals (CIs) were computed at a 95% confidence level, and a p-value of < 0.05 was considered statistically significant. All analyses were performed using R version 4.4.3 [12].

### Ethics

This study was based exclusively on publicly available, aggregated statistical data obtained from the open database of the Swedish National Board of Health and Welfare (Socialstyrelsen) and SSAR. No individual-level data were accessed or processed. Under the Swedish Ethical Review Act (SFS 2003:460), ethical review is required for research involving the processing of personal data as defined by the EU General Data Protection Regulation. As this study used only de-identified population-level statistics that do not constitute personal data, ethical approval was not required and no application was submitted to the Swedish Ethical Review Authority (Etikprövningsmyndigheten). Informed consent was not applicable, as no individual participants were included and no individual-level data were collected or processed.

## Results

### Descriptive data and patients

A total of 28,632 primary shoulder arthroplasties were performed from 2008 to 2023. Women underwent the majority of surgeries (*n* = 18,569), compared to men (*n* = 10,063). The highest number of surgeries occurred in ages 70–74 years (*n* = 5,905) and 75–79 years (*n* = 5,699). Women consistently underwent more procedures than men (Table 1).

**Table 1:**
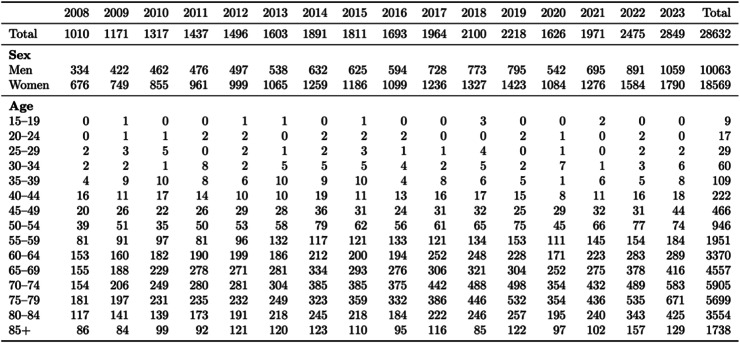
Annual number of patients undergoing primary shoulder arthroplasty by sex and age group during 2008 to 2023

### Sex and age analysis

**Fig. 1:**
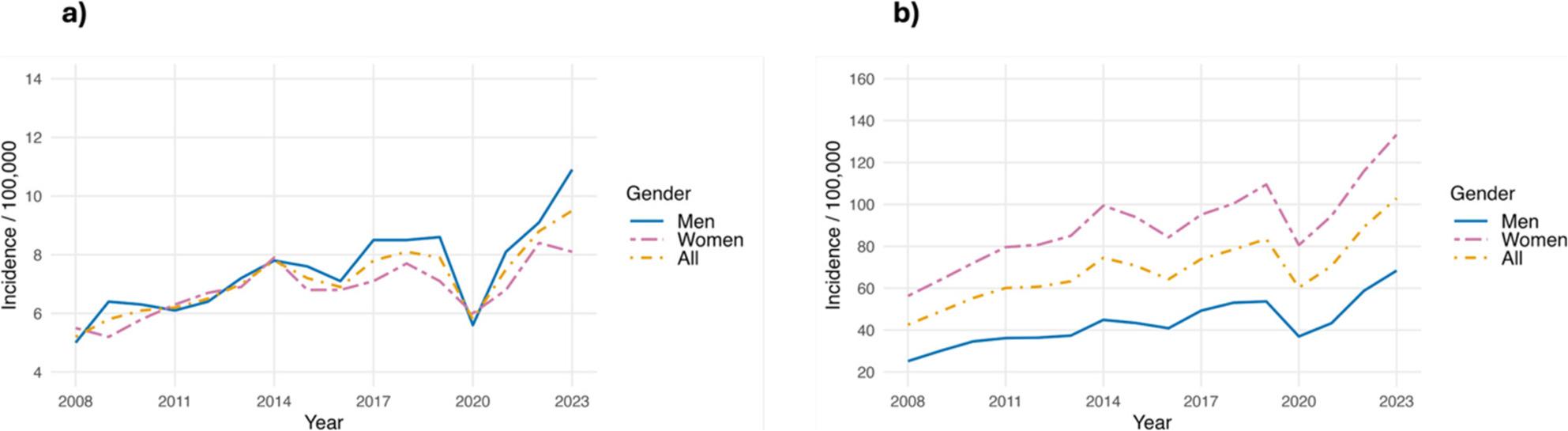
Incidence of primary shoulder arthroplasty stratified by sex during 2008–2023. (**a**) Individuals aged 15–64 years and (**b**) individuals aged ≥ 65 years

Overall, the incidence rate of primary shoulder arthroplasty increased from 13.2 to 32.7 per 100,000 during the study period (2008–2023). Women had consistently higher rates than men, trending upwards from 17.4 to 41.2. However, men showed a higher proportional increase in incidence, rising from 8.8 to 24.2. In ages 45–69 the incidence roughly doubled and almost tripled for ages 70–84 (Table  2). In ages 15–64, men showed higher incidence rates than women by the end of the study period (Fig. 1).

**Table 2:**
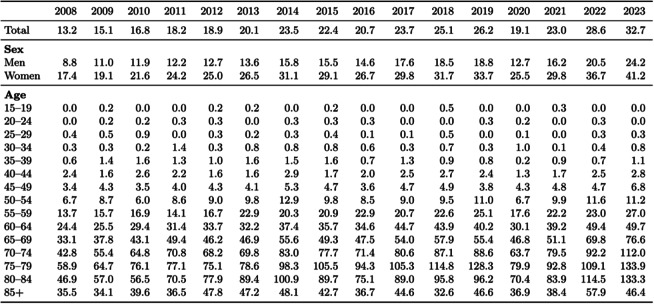
Incidence rates per 100,00 for primary shoulder arthroplasty by sex and age group during 2008 to 2023

### Surgical method analysis

**Fig. 2:**
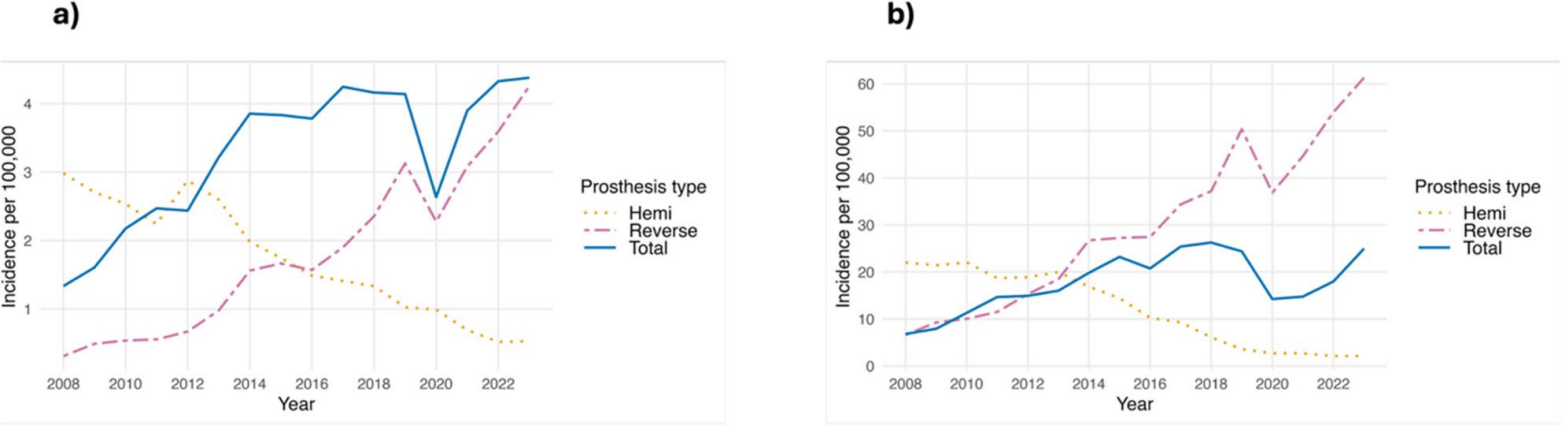
Incidence of primary shoulder arthroplasty in Sweden, 2008–2023. Panels (**a**-**b**) show incidence rates per 100,000 inhabitants by surgical method (HA, TSA, RTSA). Panel (a) includes individuals aged 15–64 years and (b) individuals aged ≥ 65 years

Usage of HA declined until it became the least used method in both age groups. Rates of TSA increased, reaching peak usage in 2018 among individuals aged ≥ 65. By 2023, TSA and RTSA were used at similar rates in the 15–64 age group. For individuals aged ≥ 65, RTSA became the most used method, with an incidence rate of 61.3 in 2023, compared to 25.0 for TSA the same year (Fig. 2).

### Indication analysis

**Fig. 3:**
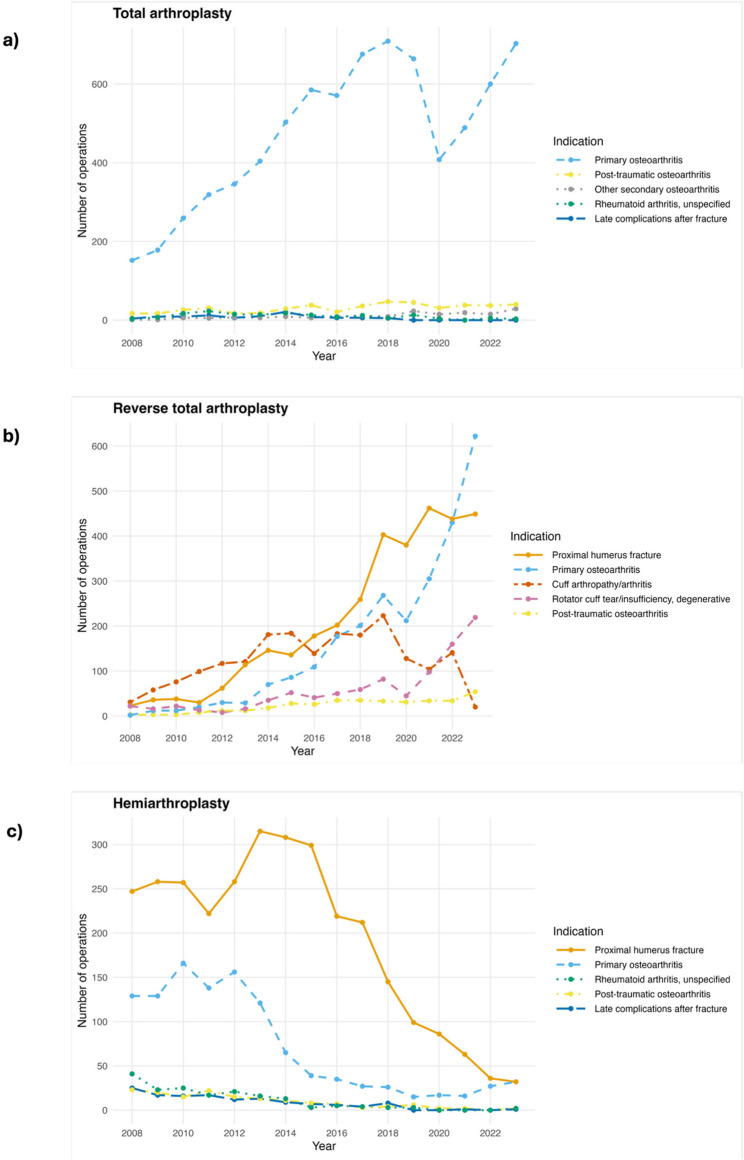
Number of primary shoulder arthroplasties by indication and prosthesis type, Panels (**a**) show the five most common indications for total shoulder arthroplasty, (**b**) reverse total shoulder arthroplasty, and (**c**) hemiarthroplasty

Primary osteoarthritis was the main indication for TSA throughout the study period. For HA, proximal humerus fracture was the leading indication, peaking in 2013 before declining towards 2023. For RTSA, proximal humerus fractures and cuff arthropathy were the most common indications in the early study period. From 2018, primary osteoarthritis increased and surpassed proximal humerus fracture volumes by 2023 (Fig. 3).

### Fixation method analysis

**Fig. 4:**
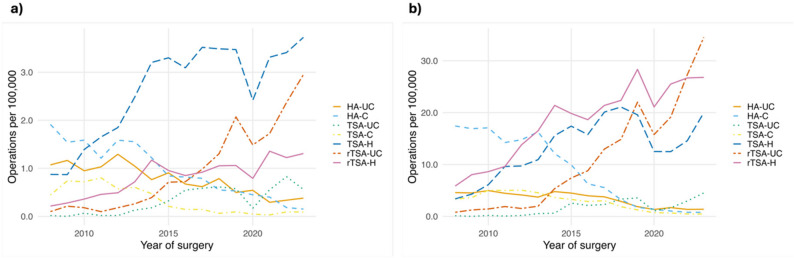
Incidence rates per 100,000 inhabitants by fixation method. Panel (**a**) shows individuals aged 15–64 years and panel (**b**) shows individuals aged ≥ 65 years. HA = hemiarthroplasty; TSA = total shoulder arthroplasty; rTSA = reverse total shoulder arthroplasty; UC = uncemented; C = cemented; H = hybrid

Cemented fixation declined at similar rates in both age groups. Uncemented and hybrid fixation were increasingly used. Hybrid fixation in TSA was the most common method of fixation among individuals aged 15–64 whereas uncemented RTSA became the most used method among individuals aged ≥ 65 years (Fig. 4).

### Future trend prediction

**Fig. 5:**
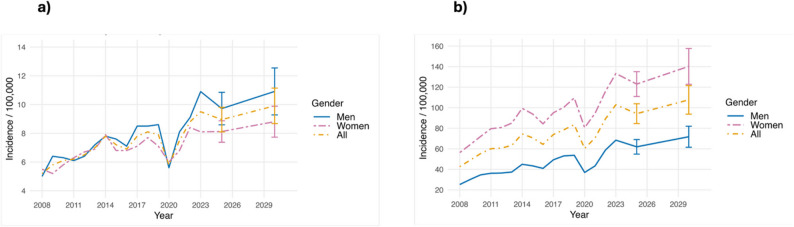
Incidence projection of primary shoulder arthroplasty stratified by sex during 2008–2023. **a** Individuals aged 15–64 years and (**b**) individuals aged ≥ 65 years

Future trend analysis of primary shoulder arthroplasty shows an upward trajectory through 2030. The overall rate is projected to rise to 9.91 per 100,000 in ages 15–64 and 108 per 100,000 in ages ≥ 65 by 2030 (Fig. 5).

## Discussion

This Swedish nationwide registry-based study shows that the incidence of primary shoulder arthroplasty more than doubled between 2008 and 2023. These trends are consistent with registry studies from other Western countries [3, 8, 13, 14]. This may reflect an ageing population with higher prevalence of shoulder osteoarthritis and cuff tear arthropathy [3, 7, 15]. As with prior findings, women aged ≥ 65 years consistently underwent more procedures than men [8, 13]. This is possibly explained by higher prevalence of shoulder osteoarthritis, rheumatoid arthritis, cuff arthropathy and proximal humerus fractures amongst women [16–20].

RTSA became the most common procedure for individuals aged ≥ 65 and was used at rates similar to TSA in individuals aged 15–64. The observed increase may be attributable to the progressive expansion of surgical indications. Initially reserved primarily for rotator cuff tear arthropathy, RTSA is now routinely used for proximal humerus fractures, glenohumeral osteoarthritis, inflammatory arthropathies and irreparable rotator cuff tears [21, 22]. Our study reports similar results. Cuff arthropathy was initially the most common indication for RTSA before primary osteoarthritis and proximal humerus fractures became the leading indications for surgery (Fig. 3).

The decline in cemented fixation is consistent with the reduced use of HA, where cemented humeral stems have traditionally been favored [23, 24]. Among individuals aged 15–64, hybrid TSA was the most common fixation type, reflecting the standard TSA construct of a cemented glenoid and an uncemented humeral stem [25, 26]. For individuals aged ≥ 65, uncemented RTSA and hybrid RTSA were most common. Increased rates of hybrid RTSA may be linked to its usage in the treatment of proximal humerus fractures [27]. The rise in uncemented RTSA across both age groups coincides with the concurrent increase in degenerative indications for RTSA and may in part be explained by this shift (Figs. 3 and 4). Comparable clinical and radiological outcomes with cemented fixation have supported a broader transition towards uncemented humeral stem fixation in RTSA [24].

Future projections suggest that by 2030, the incidence of primary shoulder arthroplasty will continue to increase, consistent with previously reported trends [28]. Women are projected to continue being the most represented group among individuals aged ≥ 65 whilst men are expected to undergo higher rates of procedures in ages 15–64. Notably, a growing body of evidence supports initial non-operative management for displaced proximal humeral fractures [29, 30], raising concerns that the growing use of RTSA in fracture management may not be fully warranted. Given the evolving landscape for surgical methods and shifts in indications, future studies are needed.

### Limitations

This study has certain limitations. Its retrospective nature as well as aggregated data limits causality. The absence of patient factors such as cuff function, disability, range of motion and comorbidity restricts further understanding of the observed trends. Finally, while future projections are based on robust historical data, they are inherently assumptions, reliant on continuation of current trends.

## Conclusion

The overall incidence rate of primary shoulder arthroplasty in Sweden more than doubled from 2008 to 2023. RTSA gained massive popularity while HA became the least used method. Women had the highest incidence rates among older individuals, while men underwent more procedures in ages 15–64. Cemented fixation declined across both age groups. Hybrid TSA and uncemented RTSA became the most common methods of fixation in both age groups. Future projections suggest a continued increase in primary shoulder arthroplasty incidence by 2030.

## Data Availability

The datasets can be obtained from the NPR directly ( https://www.socialstyrelsen.se/en/statistics-and-data/statistics/statistical-databases/ ).Reports from SSAR can be accessed through ( https://www.ssar-rapport.se/SAAR_web/publicReport.html?category=axel# ).

## Abbreviations

HA: Hemiarthroplasty
ICD-10: International Classification of Diseases, 10th Revision
NOMESCO: Nordic Medico-Statistical Committee
NPR: National Patient Register
RTSA: Reverse total shoulder arthroplasty
SNBHW: Swedish National Board of Health and Welfare
SSAR: Swedish Shoulder Arthroplasty Register
TSA: Total shoulder arthroplasty
